# Endogenous CRISPR-Cas Systems in Group I *Clostridium botulinum* and *Clostridium sporogenes* Do Not Directly Target the Botulinum Neurotoxin Gene Cluster

**DOI:** 10.3389/fmicb.2021.787726

**Published:** 2022-02-09

**Authors:** Travis G. Wentz, Benjamin J. M. Tremblay, Marite Bradshaw, Andrew C. Doxey, Shashi K. Sharma, John-Demian Sauer, Sabine Pellett

**Affiliations:** ^1^Microbiology Doctoral Training Program, University of Wisconsin–Madison, Madison, WI, United States; ^2^Division of Microbiology, Center for Food Safety and Applied Nutrition, Food and Drug Administration, College Park, MD, United States; ^3^Department of Bacteriology, University of Wisconsin–Madison, Madison, WI, United States; ^4^Department of Biology, University of Waterloo, Waterloo, ON, Canada; ^5^Department of Medical Microbiology and Immunology, University of Wisconsin–Madison, Madison, WI, United States

**Keywords:** CRISPR-Cas, CRISPR, *botulinum*, botulinum neurotoxin, conjugative plasmids, horizontal gene transfer, *Clostridium botulinum*, *Clostridium sporogenes*

## Abstract

Most strains of proteolytic group I *Clostridium botulinum* (G1 *C. botulinum*) and some strains of *Clostridium sporogenes* possess genes encoding botulinum neurotoxin (BoNT), a potent neuroparalytic agent. Within G1 *C. botulinum*, conserved *bont* gene clusters of three major toxin serotypes (*bont*/A/B/F) can be found on conjugative plasmids and/or within chromosomal pathogenicity islands. CRISPR-Cas systems enable site-specific targeting of previously encountered mobile genetic elements (MGE) such as plasmids and bacteriophage through the creation of a spacer library complementary to protospacers within the MGEs. To examine whether endogenous CRISPR-Cas systems restrict the transfer of *bont* gene clusters across strains we conducted a bioinformatic analysis profiling endogenous CRISPR-Cas systems from 241 G1 *C. botulinum* and *C. sporogenes* strains. Approximately 6,200 CRISPR spacers were identified across the strains and Type I-B, III-A/B/D *cas* genes and CRISPR array features were identified in 83% of the strains. Mapping the predicted spacers against the masked strain and RefSeq plasmid dataset identified 56,000 spacer–protospacer matches. While spacers mapped heavily to targets within *bont*(+) plasmids, no protospacers were identified within the *bont* gene clusters. These results indicate the toxin is not a direct target of CRISPR-Cas but the plasmids predominantly responsible for its mobilization are. Finally, while the presence of a CRISPR-Cas system did not reliably indicate the presence or absence of a *bont* gene cluster, comparative genomics across strains indicates they often occupy the same hypervariable loci common to both species, potentially suggesting similar mechanisms are involved in the acquisition and curation of both genomic features.

## Introduction

Botulinum neurotoxins are potent proteinaceous toxins that are horizontally distributed throughout multiple species of *Clostridium*, a genus of anaerobic, Gram-positive bacteria (Collins and East, 1998). Species differ in the serotypes they produce and serotypes vary in their ability to cause the disease botulism in humans. Eight antigenically distinct botulinum neurotoxin (BoNT) serotypes (A–G, X) have been identified in certain strains across multiple *Clostridium* species, including four species groups of *C. botulinum* (G1–4), *C. sporogenes*, *C. butyricum*, and *C. baratii* (Collins and East, 1998; Mansfield et al., 2015, 2019; Zhang et al., 2017, 2018; Brunt et al., 2018, 2020b; Contreras et al., 2019). Human botulism, systemic flaccid paralysis caused by BoNT-mediated blockade of neurotransmitter release at the neuromuscular junction, is caused by BoNT serotypes A, B, E, and F (Johnson and Montecucco, 2008). The primary BoNT serotypes produced by proteolytic group I *Clostridium botulinum* (G1 *C. botulinum*) strains are BoNT A/B/F and can occur within a conjugative plasmid or as part of a chromosomally integrated genomic island (Brunt et al., 2020a). In contrast, *C. sporogenes* only produces BoNT/B despite being the nearest neighbor species to G1 *C. botulinum* with ∼93% shared nucleotide identity between the two species. The *bont/B* gene is generally plasmid-borne in most toxigenic *C. sporogenes* strains (Weigand et al., 2015; Brunt et al., 2020a). Phylogenetic analysis based on whole genome assemblies indicates non-toxic G1 *C. botulinum* strains are rare while non-toxic *C. sporogenes* strains are relatively common (Brunt et al., 2020a). Despite G1 *C. botulinum* being responsible for a significant portion of foodborne botulism cases and being the predominant source of infant botulism due to colonization of the infant’s intestine by the toxin-producing bacteria (Arnon et al., 1979; Nevas et al., 2005), numerous questions remain regarding the means of, and restrictive barriers to, the horizontal transfer of the *bont* gene cluster in these two species. The *bont* gene carrying plasmid pCLJ from G1 *C. botulinum* has been experimentally transferred via conjugation to *C. sporogenes*, *C. butyricum*, and G3 *C. botulinum* (Marshall et al., 2010; Nawrocki et al., 2018), demonstrating inter- and intra-species plasmid transfer can occur. While plasmids are the primary *bont* associated mobile genetic element (MGE) in most *bont*(+) species, phage carry the *bont/C* and *D* genes in G3 *C. botulinum* (Eklund and Poysky, 1974). The *bont/C* and *D* genes in G3 *C. botulinum* reside within a prophage region that has been experimentally cured, ablating toxicity (Eklund and Poysky, 1974). Similar dynamics have not been observed in other species groups. While prophage have rarely been identified near specific *bont* insertion sites in several G1 *C. botulinum* strains (Smith et al., 2021b), there is no additional evidence that currently suggests phage as a driver of the propagation of the *bont* virulence factor in G1 *C. botulinum* or *C. sporogenes*.

In G1 *C. botulinum*, the mechanism by which chromosomal *bont* gene clusters are established remains unknown. However, conserved integration sites have been identified across strains and several types of MGE have been observed nearby. Several recurring integration sites for *bont* gene clusters have been identified within G1 *C. botulinum*. Within the chromosome, these include sites within *arsC*, *pulE*, disrupting the respective genes, and sites near *brnq* (Hill et al., 2009, 2015; Dover et al., 2013; Smith et al., 2021b). Several *bont* gene clusters are integrated at distinct sites. The chimeric *bont/FA(H)* gene cluster occurs at a non-standard insertion site and possesses several unique characteristics (Dover et al., 2014; Gonzalez-Escalona et al., 2014) and the unique *bont/X* gene cluster is integrated between a putative chitinase (RSJ2_770) and copper chaperone (RSJ2_773). Together, the *arsC*, *brnq*, and chitinase sites occur within 90 kbp of each other and together account for the vast majority of chromosomally integrated *bont* gene clusters. Insertion sequences (IS) are frequently present in the vicinity of *bont* gene clusters and are also a candidate for *bont* propagation. Alone, IS are simple MGEs, under 2.5 kbp and in possession of the bare minimum gene contingent necessary to facilitate their insertion and sometimes excision from a genomic site (Chandler and Mahillon, 2002). IS flanked genomic regions may become co-mobilized as a composite transposon (Siguier et al., 2014). In addition, some IS elements are adept at causing genomic rearrangements and deletions (Vandecraen et al., 2017). A recent study has demonstrated that ISs potentially play a major role in transferring virulence associated genes from conjugative plasmids to the chromosome (Che et al., 2021). Both intact and degraded ISs are known to occur within the vicinity of and, in some cases, flanking *bont* gene clusters (Smith et al., 2007, 2015; Hill et al., 2009; Dover et al., 2014). However, IS activity has not been experimentally validated in *C. botulinum* and other *bont* gene cluster carrying *Clostridia*, and it remains unknown whether IS play a role in horizontal *bont* gene cluster mobilization. Regardless of genomic localization, the ∼3.9-kbp *bont* gene is adjacent to a catalytically inactive ∼3.6-kbp paralog non-toxic non-hemagglutinin (*ntnh*) and either the *hemagglutinin* genes (*ha-33*, *ha-17*, *ha-70*) or *p47/orfX* genes (*p47*, *orfX1–3*) (Tsuzuki et al., 1990; Willems et al., 1993; Henderson, 1996), forming the *bont* gene cluster. The gene cluster ∼11–14 kbp effectively constitutes the minimal transferrable unit that an integrative mechanism would need to be able to accommodate to introduce the toxin inside the chromosome in G1 *C. botulinum*. Existing studies have predominantly focused on the chromosomal integration sites associated with *bont* genes rather than on horizontal gene transfer on a genome-wide level. Comparative genomics can be leveraged to gain additional resolution regarding whether an examined horizontal gene transfer event is species, lineage, or strain specific.

In their role as host adaptive immune modules, clustered regularly interspaced short palindromic repeats and CRISPR-associated protein (CRISPR-Cas) systems can be used to gain direct insight into horizontal gene transfer events. CRISPR-Cas systems, composed of CRISPR spacer arrays and *cas* gene clusters, are present in a wide range of bacterial species and enable the hosts to engage in sequence-specific targeting and cleavage of DNA and/or RNA (Van Der Oost et al., 2014). CRISPR-Cas systems are utilized by the bacterial host in adaptive immune and regulatory roles (Bhaya et al., 2011). In the former role, transcribed CRISPR arrays are processed by Cas6 into CRISPR RNAs (crRNA) consisting of a direct repeat and a spacer, which generally complement a fragment of foreign DNA encountered at some point in the past and initiates degradation of recognized invading DNA (Carte et al., 2008; Makarova et al., 2015).

Several bioinformatic studies have investigated CRISPR-Cas systems in G1 *C. botulinum* to varying degrees and, to the best of our knowledge, none have investigated *C. sporogenes*. As part of a survey on CRISPR-Cas systems in pathogenic bacteria, Hatoum-Aslan and Marraffini reported 14/14 closed *C. botulinum* genomes possessed type III-B CRISPR-Cas systems (Hatoum-Aslan and Marraffini, 2014). A 2017 report indicated the presence of type I-B and III-B systems in G1-3 *C. botulinum* (Negahdaripour et al., 2017). Finally, a comparative genomics study investigating recombination events at *cas* gene clusters additionally identified the presence of a type III-D CRISPR-Cas system in a subset of strains (Puigbò et al., 2017). Type I systems are generally composed of the structural proteins Cas5, Cas8, Cas7, and nuclease Cas3, and require the presence of a short protospacer adjacent motif in addition to the protospacer for cleavage of DNA to occur (Sinkunas et al., 2011; Makarova et al., 2015). Type III systems are composed of structural proteins Csm (III-A and III-D), Cmr (III-B and III-C), nuclease Cas10, and target DNA or RNA without a PAM requirement (Makarova et al., 2015; Plagens et al., 2015; Samai et al., 2015). In instances where a bacterial host possesses both type I and III systems, processed spacers may be shared between the systems and have been observed to provide functional redundancy against viral escape mutants (Silas et al., 2017). Finally, Cas1, 2, and 4 gene products have roles associated with the generation and insertion of new spacers derived from recently encountered mobile actors (Zhang et al., 2012; Nuñez et al., 2014; Lee et al., 2019). As a result, even closely related strains may have vastly different spacer arrays depending on what plasmids or phage were encountered by that strain in the environment. While CRISPR-Cas systems are generally employed against large MGEs such as plasmids and prophage, some strains of bacteria such as *Porphyromonas gingivalis* have been reported to employ CRISPR-Cas systems against IS elements, which are highly active within this species and play a major role in inter-strain diversification (Watanabe et al., 2013).

CRISPR arrays represent a library of past encounters with horizontally mobile entities including phage, plasmids, and other MGEs. Previous studies have identified type I and type III CRISPR-Cas systems in a selection of G1 *C. botulinum* strains, and none have examined their presence in *C. sporogenes* (Hatoum-Aslan and Marraffini, 2014; Carter et al., 2016; Woudstra et al., 2016; Negahdaripour et al., 2017). The number of G1 *C. botulinum* strains with fully sequenced genomes has more than doubled since these analyses, now enabling comprehensive analyses of CRISPR-Cas systems and the library of past encounters left by the systems within the genomes. We sought to examine whether the *bont* gene cluster, itself horizontally distributed, was targeted by CRISPR-Cas systems directly or indirectly through associated MGEs including plasmids, bacteriophage, ISs, or group II introns. Our in-depth analyses of 241 G1 *C. botulinum* and *C. sporogenes* strains indicate that the two species possess and utilize the same types of CRISPR-Cas systems, which do not directly target the *bont* gene clusters but target *bont*(+) conjugative plasmids.

## Materials and Methods

### Strain Selection and Phylogenetic Analysis

All available *Clostridium* refseq strains as of 3/1/2021 were downloaded from NCBI/GenBank and typed via an established MLST scheme for G1 *C. botulinum* and *C. sporogenes* (G1C)^1^ (Jacobson et al., 2008; Jolley and Maiden, 2010; Jolley et al., 2018). The pangenome and a core genome SNP (cgSNP) phylogenetic tree of 250 refseq annotated *Clostridium* genomes were determined and constructed via PanX on default settings using refseq annotations (O’Leary et al., 2016; Ding et al., 2018). Following examination of the cgSNP phylogenetic tree, eight strains of *C. sporogenes* and one unnamed strain (GCF_001276215.1, GCF_011015155.1, GCF_011016125.1, GCF_011017215.1, GCF_011017365.1, GCF_011019515.1, GCF_011020825.1, GCF_011021555.1, GCF_011021645.1) were withheld from analysis due to extreme observed distance from the major *C. sporogenes* and G1 *C. botulinum* groups. The resulting dataset consisted of 241 *Clostridium* strains [146 *C. botulinum* (including one assembly of *C. combessii*), 95 *C. sporogenes*]. BoNT serotype and subtype were determined through alignment via ClustalΩ (default, automatic) and phylogenetic analysis via raxml (-PROTGAMMAAUTO) (Sievers et al., 2011; Stamatakis, 2014; Supplementary File 1). All strains were passed through RFPlasmid (*Clostridium* model) to obtain predictions of chromosomal or extrachromosomal origin at the contig level (Van Bloois et al., 2020; Supplementary File 2).

### CRISPR Spacer Array and *cas* Gene Prediction

**FIGURE 1:**
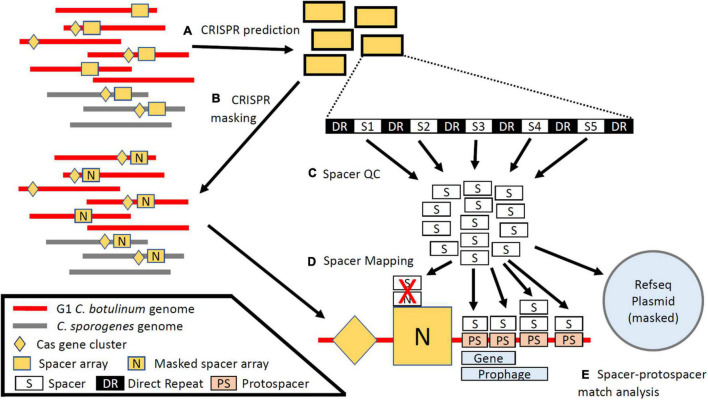
Schematic of Cas and CRISPR array identification and protospacer prediction. **(A)** CRISPR arrays and Cas genes were predicted from 241 contig-level RefSeq assemblies of group I *C. botulinum* and *C. sporogenes*. **(B)** Predicted CRISPR arrays were used to mask the assemblies and the RefSeq plasmid datasets. **(C)** Spacers were parsed by length, array length, and distance to end of the contig. **(D)** Spacers were mapped back to the masked genomes and the RefSeq plasmid dataset to identify cognate protospacer sequences. **(E)** Spacer–protospacer matches were analyzed to determine protospacer localization and predicted function.

CRISPR arrays and *cas* genes for all assemblies were predicted via CRISPR-Cas finder under default settings with Cas subtyping enabled (Figure 1A; Couvin et al., 2018). Identified *cas* genes were directly examined within the assembly for *cas* gene clusters based on established CRISPR-Cas system families (Makarova et al., 2015, 2018). Type I-B was coded as complete (I-B) if possessing cas5/6/7/8/3 and cas1/2/4 and a conserved subset possessing only cas5/6/7/8 were typed as I-B*. An additional subset of type I-B strains (I-B^**^) that were cas5/6/7/8/3(+) and cas1/2/4(−) were identified via examination of the pan-genomic data. Type III *cas* systems were coded as such if in possession of non-pseudogenized majority of the genes associated with Type III-A (cas6/10, csm 2/3/4/5/6), III-B (cas6/10, cmr1/3/4/5/6), and III-D (cas10, csm2/3/5, csx10). All detected CRISPR arrays, and 1,000 bp upstream and downstream flanking them, were utilized to mask direct to array self-matches (Figure 1B). Duplicate spacers predicted from the same assembly were dropped and spacers were assigned unique and non-redundant identifiers based on exact, directional sequence. To select for high-quality spacers and reduce spurious hits, spacers were parsed for those between 20 and 70 nt, part of an array containing five or more spacers, and not predicted within 1,000 bp of the end of a contig (Figure 1C). Parsed spacers were mapped as short reads by bowtie2 as a local alignment with allowance of up to two mismatches (-a –local -D 20 -R 3 -N 1 -L 20 -i S,1,0.5 –no-unal –no-sq –no-hd –mp 4,4 –ma 2) against protospacer datasets including Refseq Plasmid (March 1, 2021) and the 241 assemblies making up the investigative dataset (Figure 1D; Langmead and Salzberg, 2012). In addition, a subset of spacers erroneously predicted from a family of leucine-rich repeat proteins were excluded from analysis. BEDTools was utilized to obtain relevant annotations overlapping the matched protospacer sites (Quinlan and Hall, 2010). Accessions were collected for matched protospacers occurring within protein coding sequences (CDS) and functionally annotated with cluster of orthologous groups of proteins (COG) domains via the eggnog-mapper (Figure 1E) (Tatusov, 2000; Huerta-Cepas et al., 2017). Based on visual inspection of mapped results, 10 additional regions containing arrays missed by CRISPR-Cas finder prediction in certain strains were also masked (Quinlan and Hall, 2010). The plasmid NC_025146.1 was treated as part of the GCF_000829015.1 assembly for all analyses (Ihara et al., 2003; Zhang et al., 2017). Summary statistics for *cas* and spacer analysis and the complete Sp-PS match set is provided in the Supplementary Files 1, 3.

### Visualization of Spacer–Protospacer Interactions

To estimate the degree of overlap between protospacers present in both G1 *C. botulinum* and *C. sporogenes*, each spacer and protospacer was grouped by species clade. The resulting species-spacer and species-protospacer groups and their associated unique CRISPR spacer IDs were treated as node-edge pairs in a directed force matrix. Node pairs were visualized via Cytoscape and grouped using the yFiles organic layout (Shannon, 2003). The resulting force matrix was colored by spacer clade (G1 *C. botulinum*: green, *C. sporogenes*: blue) and organized such that spacers mapping to protospacers present in both species are centrally located within the diagram, spacers mapping to protospacers present in only one clade at the top and bottom, and unmatched spacers fanning out from the spacer species. Darker edge coloration indicates greater edge density.

### Additional Investigation of *Clostridium sporogenes* str. CDC 1632 and Analysis of *ltrA* Distribution

Mauve was used to initially align plasmid pNPD7 (NZ_CP013241.1) and the *C. sporogenes* str. CDC 1632 chromosome (Darling, 2004). Prophage regions were located, scored, and annotated via PHASTER (Arndt et al., 2016). BlastN, default settings, was used to generate local alignment data between the CDC 1632 putative integrated plasmid (coordinates: 3983434–4238643 bp) and pNPD7 (Altschul et al., 1990). The *ltrA* CDSs identified in *C. sporogenes* str. CDC 1632 were provided as a tblastn query against the 241 strains in the dataset (*E* value 1e-5, word size 2) (Supplementary File 4). BlastN, default settings, was used to align a portion of S2 from G1 *C. botulinum* str. 1169 and S6 from G1 *C. botulinum* str. A3 Loch Maree, and matches less than or equal to 1E-50 were visualized via Kablammo and gene cluster diagrams were generated via Gene Graphics (Altschul et al., 1990; Wintersinger and Wasmuth, 2015; Harrison et al., 2018). Representative *ltrA* sequences were aligned via Clustal Omega, default settings (Rice et al., 2000; Sievers et al., 2011). The secondary structure of the catalytic RNA structure flanking *ltrA* (WP_012300946.1) in *C. botulinum* str. A3 Loch Maree was predicted via the MXfold server, and domains were manually annotated through consultation of the group II intron database (Candales et al., 2012; Sato et al., 2021). All phylogenic tree graphics were built via iTOL (Letunic and Bork, 2019). The program phylocorrelate was run in conjunction with the cgSNP tree to investigate correlation between identified group II introns, *bont* genes, and pfam annotated protospacers (Supplementary File 4).

### Analysis of *bont* and *cas* Integration Sites

Through investigation of the literature and analysis of the predicted *bont* and *cas* gene loci, seven distinct loci encompassing all known sites of *bont* and *cas* gene cluster integration were identified within the subset of complete/closed genomes (*n* = 43/241). Prophage regions were identified for each chromosome via PHASTER (Arndt et al., 2016). Stable flanking genes were identified for each chromosomal and plasmid site associated with *bont* and/or *cas* gene cluster features. Chromosomal sites were defined as S1: *cysK-brnQ*, S2: *arcA-ytaF*, S3: *efp-cloSI*, and S4: *bglG-*αβ*-hydrolase*; plasmid sites as S5: *dnaX*-*ATPase*, S6: *viralA*-*thermonuclase*, and S7: *DUF1292-DUF3854*. Site loci for each genome are provided in Supplementary File 5. The closed genomes for *Clostridium botulinum* str. Mfbjulcb8 (genetic *C. sporogenes*) and G1 *C. botulinum* strain 1169 were excluded from analysis due to a unique chromosomal rearrangement that disrupted the insertion sites and a PHASTER prediction error, respectively. Spacers overlapping an annotated site or phage were assigned corresponding codes, and all others were assigned to the chromosome or plasmid. Only chromosomes and *bont*(+) plasmids associated with the 43 closed genomes were included in the analysis.

### Statistical Analysis of Protospacer Density Across Closed Genomes

A protospacer density metric was calculated as the number of protospacer loci divided by feature length in base pairs for prophage, plasmid, and chromosomal features in the 43 strains. For determination of chromosomal protospacer density, chromosomal prophage regions and associated protospacers were subtracted from chromosome length and protospacer count. Five plasmids (NCBI Accession: NZ_CP014147.1, NZ_CP013848.1, NZ_CP014218.1, NZ_CP014173.1, NZ_CP031100.1) were classified as phage following observation of numerous structural bacteriophage proteins throughout the length of the plasmid. Protospacer density from plasmids (*n* = 19), phage (*n* = 115), and the chromosome (*n* = 43) were normalized via log transformation [Log10(Protospacer Density) + 7] and protospacer density was assessed across groups via the Welch one-way ANOVA test in the rstatix R package (Wickham, 2011; R Core Team, 2013; Wickham et al., 2019; Kassambara, 2020, 2021). Plasmids and phage with no matched protospacers were excluded from analysis (*n* = 69). A non-parametric *post hoc* analysis (Games–Howell) was run following the ANOVA to determine statistically significant mean differences between the three feature groups (Kassambara, 2021).

## Results

### Group I *Clostridium botulinum* and *Clostridium sporogenes* Are Distinct, Closely Related Species

**FIGURE 2:**
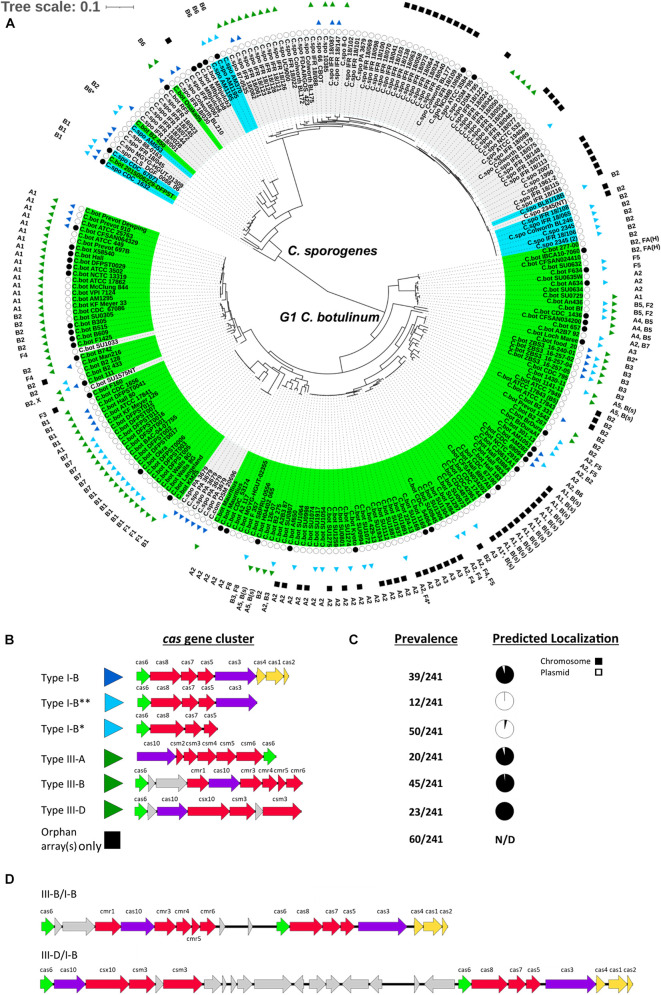
CRISPR-Cas systems in group I *C. botulinum* and *C. sporogenes*. **(A)** A core genome SNP matrix phylogenetic tree of 241 G1 *C. botulinum* and *C. sporogenes* indicating two major clades predominantly demarcated by the respective species. Closed black circles indicate complete genomes (*n* = 45). Cas gene clusters are represented by colored arrows and correspond to CRISPR-Cas system type in **(B)**. Solid black square indicates strains with predicted CRISPR arrays but no *cas* genes. Predicted BoNT serotype(s) are listed on the outermost band of the tree and strain names shaded by published species and toxin presence: *bont*(+)/G1 *C. botulinum*, *bont*(+)/*C. sporogenes*, bont(−). **(B)** Gene schematic of the major *cas* gene clusters identified in G1 *C. botulinum* and *C. sporogenes*. **(C)** Prevalence of each CRISPR-Cas system type/subtype throughout the assemblies included in the study dataset and distribution by predicted plasmid or chromosomal localization for each contig carrying the CRISPR-Cas system subtype. **(D)** Potential hybrid/coupled type I-B and type III-B or III-D CRISPR-Cas systems generally occur in strains in possession of both.

To facilitate analyses of CRISPR-Cas systems throughout G1 *C. botulinum*, we conducted a pan-genomic analysis and constructed a core genome SNP phylogenetic tree of 241 strains of G1 *C. botulinum* and *C. sporogenes* (Figure 2A). The analysis revealed 2,003 shared orthologous genes between the two strains (Supplementary Table 1), confirming two highly related species (Figure 2). This is similar to two previous studies utilizing cgSNP phylogeny approaches, which identified 2,016 and 2,420 shared orthologous genes between G1 *C. botulinum* and *C. sporogenes*, indicating distinct but closely related species (Weigand et al., 2015; Brunt et al., 2020a). The final dataset consisted of 45 closed and 196 contig level assemblies and the phylogeny split into G1 *C. botulinum* and *C. sporogenes* clades (Figure 2A and Supplementary File 1). Of the 17,472 contigs in the dataset, 1,573 contigs accounting for 2.95% of total nucleotide content were predicted as extrachromosomal (Supplementary File 2).

Consistent with prior studies, several non-toxic strains loaded within the G1 *C. botulinum* clade, including *C. sporogenes* str. PA 3679, *C. combesii*, and G1 *C. botulinum* strains SU1575NT and SU1033 (Figure 2A; Butler et al., 2017; Dobritsa et al., 2018; Brunt et al., 2020a). Conversely, seven strains previously described and deposited as *C. botulinum* loaded into the *C. sporogenes* clade, four of which possessed no detected *bont* gene cluster (Figure 2A and Supplementary File 1). Analysis of closed genomes indicated serotypes A/B/F occur as either plasmid-borne or chromosomally integrated clusters in G1 *C. botulinum* (Figure 2A and Supplementary Files 1, 2). Conversely, only 16 of the 89 *C. sporogenes* assemblies were found to carry a *bont* gene cluster and they exclusively encode BoNT/B1, B2, or B6. Among the three closed *C. sporogenes* genomes encoding *bont* clusters, two occurred on plasmids. Predicted localization of these toxin types in contig level *C. sporogenes* assemblies also favored plasmid localization (Supplementary File 2). A single strain, *C. sporogenes* CDC 1632, the most extreme outlier within the phylogeny, possessed a chromosomally integrated *bont/B1* gene cluster (Figure 2A). Taken together, these data indicate that *bont* gene clusters are broadly present throughout G1 *C. botulinum* and frequently chromosomally integrated, while *bont* gene clusters in *C. sporogenes* are limited to *bont/B* and usually plasmid-borne.

### CRISPR-Cas Systems (CRISPR-CAS System) and Features Are Broadly Distributed Throughout G1 *Clostridium botulinum* and *Clostridium sporogenes*

Similar to previous observations that type I and type III CRISPR systems are present in *C. botulinum* strains (Carter et al., 2016; Woudstra et al., 2016; Negahdaripour et al., 2017), most of the G1 *C. botulinum* and *C. sporogenes* strains examined in this study (202/241, 84%) contained one or more predicted *cas* gene clusters or an orphan CRISPR array in the absence of a *cas* gene cluster (Figure 2B). Type III-A, B, D, and Type I-B CRISPR-Cas systems possessing a defined *cas* gene cluster were found in 141 strains (Figure 2). The remaining 100 assemblies possessed no identifiable *cas* gene clusters, although a majority had at least one orphan CRISPR array present. The adaptation module genes *cas1*, *cas2*, and *cas4* (*cas1/2/4*) were only observed in association with type I-B gene clusters and were present in only 39 assemblies, indicating that the capacity to generate novel spacers is relatively rare throughout the population and exclusive to type I-B. While not uncommon for type III systems to lack the adaption module and instead rely on those associated with type I systems (Makarova et al., 2013, 2015), 67 assemblies possessed a type III system with no *cas1/2/4*(+) type I-B system present (Figure 2A and Supplementary File 1). In addition, two partial variants of the type I-B CRISPR-CAS system lacked adaption (I-B^**^) and *cas3* nuclease (I-B*) genes (Figure 2B). Since *cas6* is the only universally present gene in all investigated assemblies, we analyzed homology of this gene within our data set. Multiple alignment of annotated *cas6* indicated deep divergence at the amino acid level between the partial I-B variants and within complete I-B CRISPR-Cas systems (Supplementary Figure 1). These data indicate a diverse range and variable presence of complete and incomplete CRISPR-Cas systems throughout G1 *C. botulinum* and *C. sporogenes*, with no clear species-specific phylogenetic distinction between the observed CRISPR-Cas systems and CRISPR elements.

### In Both Species, Complete Type I-B and Type III *cas* Gene Clusters Localize to the Chromosome While Partial I-B *cas* Systems Localize to Plasmids

While primarily utilized by the bacterial host as a means of adaptive immunity, it is increasingly recognized that some MGEs, including bacteriophage, transposases, and plasmids, also possess and utilize CRISPR-Cas systems for regulatory roles and self-preservation (Faure et al., 2019; Klompe et al., 2019; Mcdonald et al., 2019; Varble et al., 2019). Analysis of the genomic localization of the *cas* gene cluster in the 141 assemblies containing them showed that 96% of complete type I-B and type III CRISPR-Cas systems localized to the chromosome or contigs predicted to be chromosomal. The remaining 4% (6 assemblies) of these types with predicted plasmid localization resided on short contigs, which impacts prediction accuracy (Van Bloois et al., 2020). These data indicate that the complete type I-B systems, the sole type identified with the potential to generating novel spacers within both species, are chromosome exclusive. In contrast, the partial type I-B CRISPR-Cas system variants localized exclusively to plasmids (Figure 2C). The partial type I-B variant, I-B^**^, localized to a family of ∼200 kbp, *bont*(-) plasmids, and I-B* to the family of *bont*(+) conjugative plasmids ∼250 kbp (Figures 2B,C). Two chromosomally localized type I-B* CRISPR-Cas systems were observed; however, analysis of the genomic regions surrounding the CRISPR-Cas systems indicated that they were localized within chromosomally integrated *bont*(+) plasmids in *C. sporogenes* str. 1632 and *C. botulinum* str. DFPST0006. These chromosomal plasmid integrations have recently been independently reported (Smith et al., 2021a). In strains where both type I-B and III-B or III-D CRISPR-Cas systems were present, the gene clusters encoding the two CRISPR-Cas systems were frequently adjacent to each other within the chromosome (Figure 2D). These data show differential localization of *cas* subtypes and in *C. botulinum* and *C. sporogenes*.

### Both *cas* and *bont* Gene Clusters Localize to Shared Sites (S1–S4) Within the Chromosome in G1 *Clostridium botulinum* and *Clostridium sporogenes*

**FIGURE 3:**
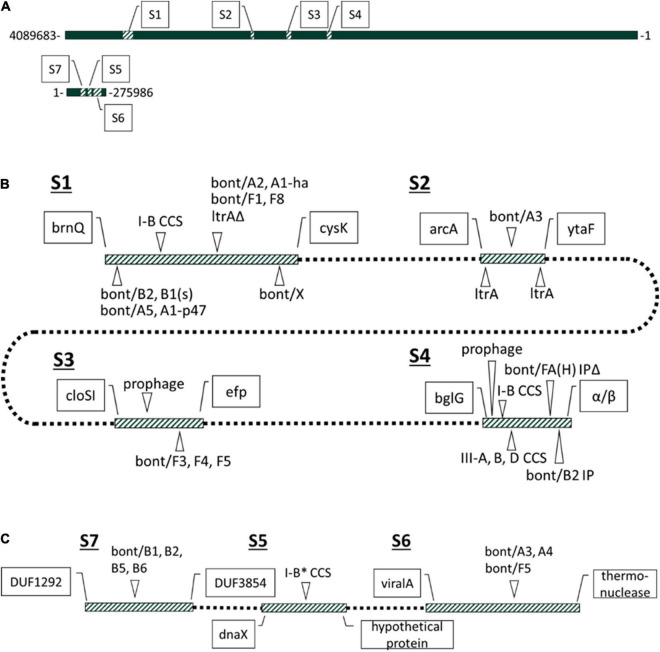
Discrete localization sites of *bont* gene cluster and CRISPR-Cas systems in *C. botulinum* and *C. sporogenes*. **(A)** Genomic locations of seven specified sites associated with *cas* and *bont* gene cluster in G1 *C. botulinum* and *C. sporogenes* host diverse MGEs. Sites annotated within the *C. botulinum* CDC_1436 genome. **(B)** Strain variable *bont*, *cas*, and MGE associated features of the four chromosomal sites known to harbor with *bont* and *cas* gene clusters. At S1, a fragment of the group II intron (*ltrA*Δ) intron-encoded protein co-occurs with *bont* gene clusters integrated at the *arsC* gene. **(C)** Plasmid sites associated with bont and *cas* gene clusters. Boundaries defined for all sites in 43 strains in Supplementary File 5.

CRISPR-Cas systems frequently occur at dynamic sites within the genome that over time can accrue additional genes of related functions such as complementary CRISPR-Cas systems, RM systems, and other genes that may play a defensive role (Makarova et al., 2011; Doron et al., 2018). Having observed some degree of positional overlap between the type III and type I-B systems in the form of adjacent/hybrid systems (Figure 2D), we further characterized the regions flanking these CRISPR-Cas systems. Within closed assemblies, type I-B CRISPR-Cas systems are present in two distinct chromosomal genomic regions, while all type III CRISPR-Cas systems localized to only one of the two chromosomal genomic regions (Figures 3A,B). Limited examination of contig-level assemblies revealed a subset of III-A CRISPR-Cas systems localized elsewhere, indicating additional CRISPR-Cas system sites may exist (Supplementary Figure 1). Analysis of the flanking region showed the *bont* gene clusters predominantly occupy sites near type I-B CRISPR-Cas systems at site 1, which encompasses *brnQ* and *arsC bont* integration sites. Two *bont* gene clusters occurred within fully or partially chromosomally integrated plasmid sequences near the CRISPR-Cas systems integration regions (Supplementary Figure 2; Dover et al., 2014; Hill et al., 2015; Smith et al., 2021a). The only *cas* gene clusters observed outside of the chromosome were cas1/2/4(−) I-B^**^ cas gene clusters exclusive to a family of *bont*(−) plasmids and nuclease(−) cas1/2/4/3 type I-B* *cas* gene clusters exclusive to *bont*(+) conjugative plasmids. The type I-B^**^ system and the *bont*(−) plasmids were not further characterized beyond that the ∼200-kbp plasmid family is distinct from and unrelated to the ∼250-kbp *bont*(+) conjugative plasmids (Supplementary Figure 2). The *bont*(+) conjugative plasmids of G1 *C. botulinum* and *C. sporogenes* were found to share large conserved regions and common elements. This indicates *bont*(+) conjugative plasmids constitute a related plasmid family (Supplementary Figure 2), which is consistent with a previous report showing relatedness between *bont/b* bearing plasmids in *C. botulinum* (Hosomi et al., 2014; Orlek et al., 2017). All type I-B* CRISPR-Cas systems localized to one site within the *bont*(+) conjugative plasmid family and were present in all family members with the exception of pCLJ and p1_CDC51232 (Figure 3C and Supplementary Figure 2). No *bont* gene clusters were detected near the type I-B* plasmid integration site, but the *bont* gene clusters on the *bont*(+) conjugative plasmid family exclusively localized to two distinct plasmid integration sites. Conserved genomic markers were identified within the vicinity of all *bont* and *cas* gene clusters within the 43 closed genomes, defining seven distinct genomic regions. Four chromosomal regions were denoted as sites 1–4 and three plasmid regions as sites 5–7 (Figures 3A,B).

Whereas S4 is the primary chromosomal CRISPR-Cas system integration site in both species and an atypical *bont* gene cluster integration site, S1 is the primary chromosomal *bont* gene cluster integration site in G1 *C. botulinum* and occasional type I-B CRISPR-Cas systems integration site. Sites 2 and 3 contain *bont* gene clusters but no CRISPR-Cas systems. Similarly, on plasmids *bont* gene cluster associated sites 6 and 7 lack CRISPR-Cas systems, while site 5 hosts the type IB* CRISPR-CAS system and no *bont* gene cluster (Figure 3C and Supplementary Figure 2).

Further analysis of integration sites within the 43 closed assemblies revealed pseudogenized *cas6* genes and orphan arrays in place of a full *cas* gene cluster in 3 strains at site 1, and in 4 strains at site 4, indicating CRISPR-Cas system degradation and loss in some lineages (Supplementary File 5). In addition, these sites are host to a variety of MGEs including ISs (Dineen et al., 2003; Hill et al., 2009) and group II introns (Figure 3B and Supplementary File 5). Comprehensive analysis of group II introns throughout the study dataset revealed full-length group II introns flanking the *bont/A3* gene cluster at S2 (Figure 3B and Supplementary Figures 3A,B). In addition, a fragment of the group II intron, intron-encoded protein is present at the site of the disrupted *arsC* gene (site S1) in all G1 *C. botulinum* strains with an *arsC* integrated *bont* gene cluster (Figure 3B and Supplementary Figure 3C). However, group II introns, like ISs, which have also been observed in the vicinity of and within the boundaries of *bont* gene clusters (Dineen et al., 2003; Dineen, 2004; Smith et al., 2015), can also be found independently of the *bont* gene cluster. Correlation analysis via phylocorrelate supported significant association only between the *ltrA*/*ltrA*-fragment and *bont*/A2, but not other *arsC*-S1 associated *bont* gene clusters (Supplementary File 4; Tremblay et al., 2021). This could be due to under/over-representation of certain *bont* subtypes in the study dataset or a true lack of correlation. Despite this, group II introns appear to be one of the more consistently present small MGEs within the vicinity of a diverse group of chromosomal and plasmid localized *bont* gene clusters.

These findings indicate that sites S1 and S4 serve as hypervariable regions that attract and accumulate MGEs and horizontally acquired cargo genes. Sites S2 and S3 lack CRISPR-Cas systems but provide examples of MGEs that occur within the vicinity of *bont* gene clusters. Site S2 was the only chromosomal integration site found for the *bont/*A3 gene cluster flanked by group II introns, and S3 contained *bont*/F3, F4, and F5 gene clusters and bacteriophage have previously been identified in the presence and absence of the *bont*/F gene cluster (Figure 3B; Smith et al., 2020). Taken together, we have shown seven chromosomal and plasmid integration sites in G1 *C. botulinum* and *C. sporogenes*, which contain CRISPR gene clusters and/or *bont* gene clusters as well as several other MGEs. This indicates genomic hotspots for integration of both defense islands as well as virulence genes and other MGEs. While the association between the *bont* gene cluster and some MGEs occurs sporadically, it is also necessary to account for CRISPR-Cas system targeting of associated MGEs which could, by proximity, limit the horizontal transfer of *bont* gene clusters.

### CRSIPR-Cas Systems of G1 *Clostridium botulinum* and *Clostridium sporogenes* Predominantly Target Plasmids and Bacteriophages but Not *bont* Gene Clusters

To investigate whether predicted CRISPR-Cas systems in G1 *C. botulinum* and *C. sporogenes* could potentially modulate the range of *bont* gene transfer through immunity by either directly targeting the *bont* gene cluster or associated MGEs, we investigated the CRISPR array encoded spacers and identified their predicted cognate targets (protospacers). We first examined the global protospacer matches identified via spacer mapping against all 241 strains and the RefSeq plasmid database. Across all assemblies, a pool of 6,208 spacers was identified. Of those, 60.4% mapped to protospacer targets present in the strain and/or RefSeq plasmid dataset (Supplementary Table 2A; Brooks et al., 2019), with a total of 55,729 spacer–protospacer matches identified. The high percentage of matched spacers reflects the stringent quality control applied to spacers, strain redundancy, and high prevalence of protospacers within the study data set. Both G1 *C. botulinum* and *C. sporogenes* genomes were heavily targeted (Supplementary Table 2A). Of all spacer matches, 26% mapped to proteins with detectable conserved COG domains (Supplementary Table 2A; Tatusov, 2000; Huerta-Cepas et al., 2017). Categorization of remaining hits by RefSeq annotation revealed 20% mapped to phage associated proteins, 16% to intergenic loci, 9% to proteins with an annotated putative function but no COG match, 4% to proteins with a domain of unknown function (DUF), and 25% to hypothetical proteins (Supplementary Table 2A). Consistent with other studies, a small fraction of spacers (*n* = 130), were self-matches within the same genome (Supplementary File 6). This low prevalence is largely consistent with the expectation that self-matches are deleterious to the host (Shmakov et al., 2020). Among protospacer matches belonging to known COG categories, the most heavily targeted proteins in both *C. sporogenes* and G1 *C. botulinum* were those relating to: (1) replication, recombination, and repair; (2) transcription; and (3) cell wall/membrane/envelop biogenesis (Supplementary Table 2B). Overall, these results are consistent with general expectations that CRISPR-Cas systems primarily target gene products that bacteriophage and plasmids require to replicate and propagate (Bhaya et al., 2011).

Investigation of protospacer annotations indicated the *bont* gene cluster is not a direct target of CRISPR-Cas systems in either species. No protospacers were identified within any of the genes of the primary *bont* gene cluster genes: *bont*, *ntnh*, *ha17*, *ha33*, *ha70*, *p-47*, *orfX1–3*, or *botR*. Very few annotated matches to IS elements were identified, namely, spacers from G1 *C. botulinum* str. B2 331 and the *C. sporogenes* PA3679 strains matched protospacers in a *tnpB* sequence of IS200/IS605-like ISs present exclusively on several predicted *bont*(+) conjugative plasmids (Supplementary File 6). No protospacers within annotated group II introns (*ltrA*) were identified. A single redundant spacer present in both *C. sporogenes* strains IFR 18/061 and IFR 18/062 was observed to match a putative peptidoglycan binding gene, which may frequently co-occur with *p47/orfX(*+) *bont* gene clusters (Smith et al., 2021b; Supplementary File 6). However, these same spacers were also observed to match other copies of this gene that occurred independently of the *bont* gene clusters. These findings suggest that, in aggregate at the species wide-scale, CRISPR-Cas systems do not represent a direct barrier to the trafficking of *bont* gene cluster genes throughout G1 *C. botulinum* and *C. sporogenes*.

**FIGURE 4:**
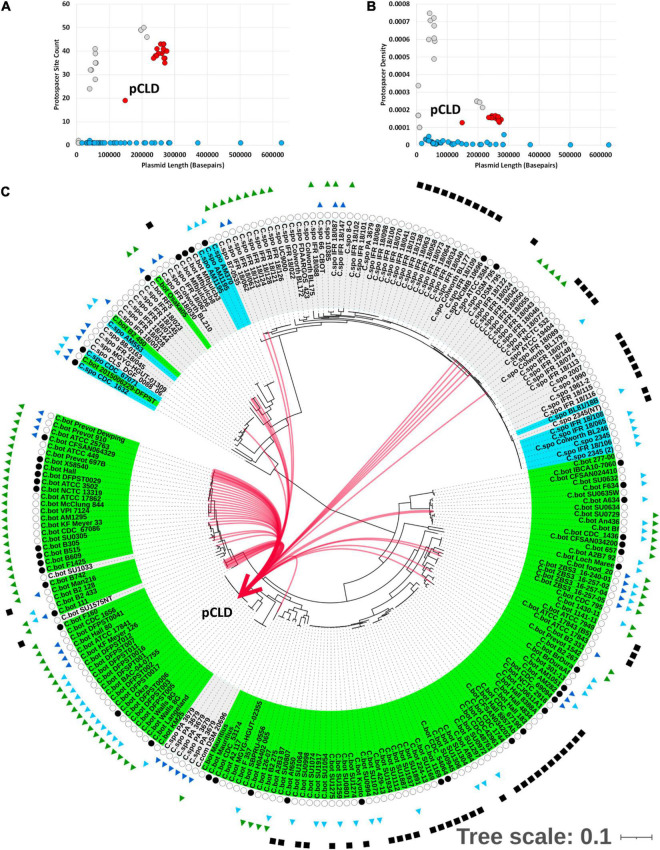
*bont*(+) conjugative plasmids targeted by CRISPR-Cas systems in G1 *C. botulinum* and *C. sporogenes*. **(A)** Plasmid length (*x*-axis) by number of (redundant) spacer–protospacer (Sp-PS) matches (*y*-axis) for Refseq plasmids; BoNT(+) plasmids in red, bont(−) plasmids from *C. sporogenes* and G1. *C. botulinum* in gray, matches to plasmids from other species in blue. **(B)** Transformation of **(A)** to plasmid length (*x*-axis) by Protospacer Density (Sp-PS matches/plasmid length) (*y*-axis) by plasmid length. **(C)** Visual representation of matched spacer containing assemblies to plasmid pCLD.

To determine whether plasmid-borne *bont* gene clusters may be indirectly targeted through targeting of the plasmid vehicle, spacer–protospacer matches to verified plasmids were assessed. Both the *bont*(+) ∼250 kbp and *bont*(−) ∼200-kbp plasmids are targeted by spacers from G1. *C. botulinum* and *C. sporogenes*, and a higher density of matches is observed relative to those in plasmids from other species (Figures 4A,B and Table 1). Despite being an outlier in the *bont*(+) plasmid family, due to the absence of a ∼100-kbp region including the predicted type IV secretion system, pCLD possesses equivalent protospacer density to the rest of the family (Figures 4A,B). These results indicate that plasmid protospacers are broadly distributed across the length of the plasmid, and protospacer profiles can be used to further characterize plasmid families. Matches involved a range of functions including replication and toxin–antitoxin matches, potentially representing examples of spacers both with generalized anti-plasmid targeting and with specific targeting of gene products representing a direct threat to host survival (Table 2). In addition, spacers with matches to pCLD come from a broad range of CRISPR-Cas system(+) assemblies including those harboring hybrid CRISPR Cas systems type I-B/III, I-B only, III only, and plasmid-borne partial I-B variants (Figure 4C). Overall, these results indicate that the *bont*(+) plasmids are targeted by a range of CRISPR-Cas systems present throughout both species. However, questions remain as to how impactful this is at the species level, considering the prevalence of *bont*(+) plasmids and CRISPR-Cas features in both species.

**TABLE 1:** Count of RefSeq plasmids and protospacer loci by species matched to spacers from the 241 strain G1 *C. botulinum* and *C. sporogenes* dataset.

Species	Matched protospacer positions	Matched plasmid count
*Acinetobacter baumannii*	1	1
*Bacillus cereus*	5	5
*Bacillus thuringiensis*	5	5
*Chroococcidiopsis thermalis*	2	1
*Clostridioides difficile*	2	2
*G1 Clostridium botulinum*	991	31
*G2 Clostridium botulinum*	2	1
*Clostridium perfringens*	1	1
*Clostridium sporogenes*	126	4
*Enterococcus avium*	1	1
*Enterococcus faecium*	12	12
*Enterococcus mundtii*	1	1
*Eubacterium eligens*	1	1
*Lactiplantibacillus plantarum*	1	1
*Methanomethylovorans hollandica*	1	1

**TABLE 2:** Annotations associated with pCLD protospacers matched to spacers derived from 44 G1 *C. botulinum* and *C. sporogenes* strains (Figure 4C).

pCLD protospacer annotations	Spacer–protospacer matches
AAA family ATPase	7
Bacitracin ABC transporter ATP-binding protein	2
DNA polymerase III subunit delta	1
Helix-turn-helix transcriptional regulator	1
Hypothetical protein	26
Intergenic	14
Methyltransferase	1
Phosphoadenosine phosphosulfate reductase family protein	1
Type II toxin-antitoxin system death-on-curing family toxin	1
Viral A-type inclusion protein	4

**FIGURE 5:**
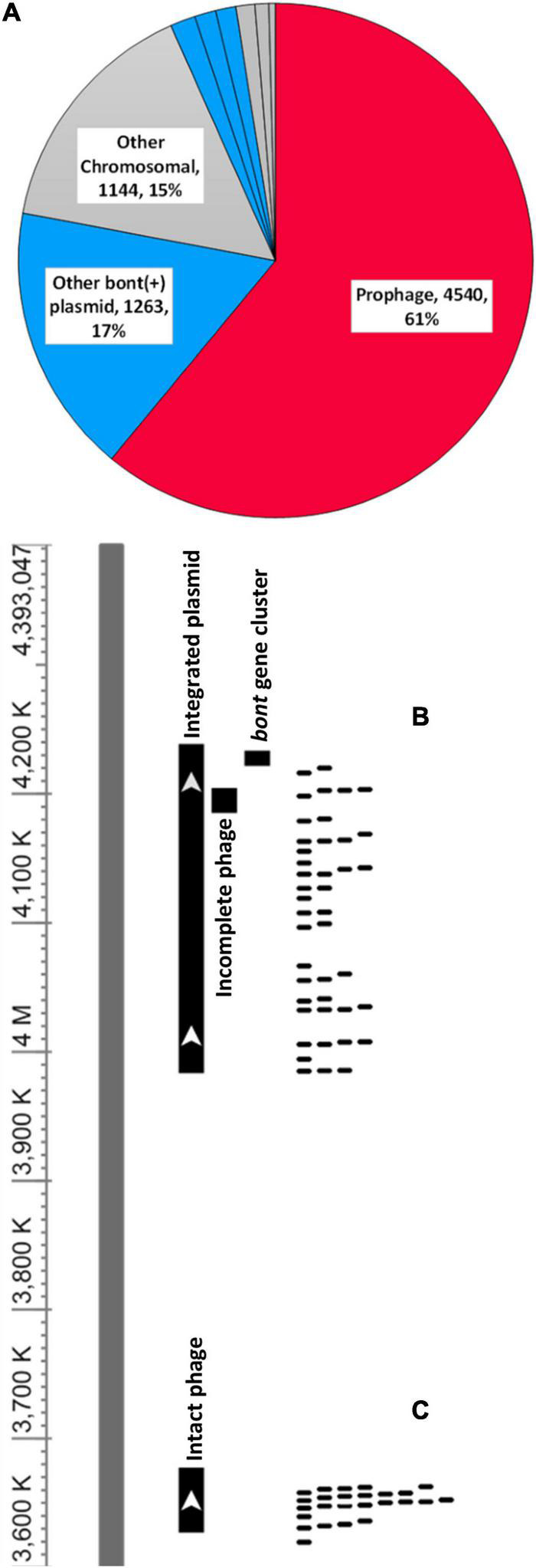
Botulinum neurotoxins are not targets of CRISPR spacers. **(A)** Pie chart showing 7,005 spacer–protospacer pairs from 43 closed genomes categorized by genomic localization. Individual sites are detailed in Table 3. Genomic sites 1–7 are defined as in Figure 3. Boundaries for each genome are provided in the Supplementary File 5. Prophage refers to any protospacer present within a chromosomal prophage region. Other *bont*(+) plasmid to any *bont*(+) plasmid protospacers falling outside the defined sites S5–7 and other chromosomal to any protospacers outside of a predicted phage or defined sites S1–4 (Figures 3B,C). **(B,C)** Protospacers occur at a higher density in prophage and plasmids relative to the chromosome. Mapping spacers to the closed genome of *C. sporogenes* CDC 1632 revealed through protospacer matches, both prophage associated regions and an integrated *bont*(+) plasmid.

The horizontal mobility of the *bont* gene cluster could also be affected through targeting of nearby and interceding genomic features. To investigate the presence of protospacers in intergenic matches within and near the *bont* gene cluster and potentially associated smaller MGEs, we globally categorized protospacers in sites S1–S7 in a subset of strains with closed assemblies. Intergenic protospacers accounted for 15 and 17% of *C. sporogenes* and G1 *C. botulinum* hits (Supplementary Table 2A). However, there are limitations in the accurate identification of promiscuous and often pseudogenized MGEs, such as group II introns and ISs, by annotation alone. To comprehensively examine potential intergenic protospacers and MGEs within and around *bont* gene clusters in an annotation agnostic way, we examined protospacer matches within the sites established in Figure 3. Of the 7,443 spacer-protospacer matches present in the closed assemblies surveyed, which included 43 chromosomes and 11 *bont*(+) plasmids, 61% were found within predicted prophage (Figure 5A). Within the defined genomic sites S1–7, protospacer matches accounted for 7% of all matches. However, no matches to *bont* gene cluster genes, group II introns, or ISs were detected (Figure 5A, Table 3, and Supplementary File 7). At site S2, which harbors the *bont*/A3 gene cluster, no protospacers were identified. Of the remainder of protospacer matches, outside of annotated bacteriophage and the seven defined sites S1–S7, 17% were found in the *bont*(+) plasmid and 15% in the chromosome. A visual pile-up representation of these dynamics in *C. sporogenes* str. CDC 1632 demonstrates the utility of this approach in the investigation of integrated plasmids and prophage (Figures 5B,C). Taken together, these results indicate that the spacer arrays in G1 *C. botulinum* and *C. sporogenes* are predominantly composed of anti-phage and anti-plasmid spacers and do not directly target *bont* gene clusters, nearby insertional elements, or group II introns.

**TABLE 3:** Proportion of protospacer hits falling within the defined *bont* and *cas* associated gene clusters.

Site	# Spacer–protospacer matches	% of total matches
S6	118	1.59
S7	99	1.33
S5	95	1.28
S4	89	1.20
S1	64	0.86
S3	31	0.42
S0	0	0.00

*No protospacers were identified with S2 (associated with Figure 5A).*

**FIGURE 6:**
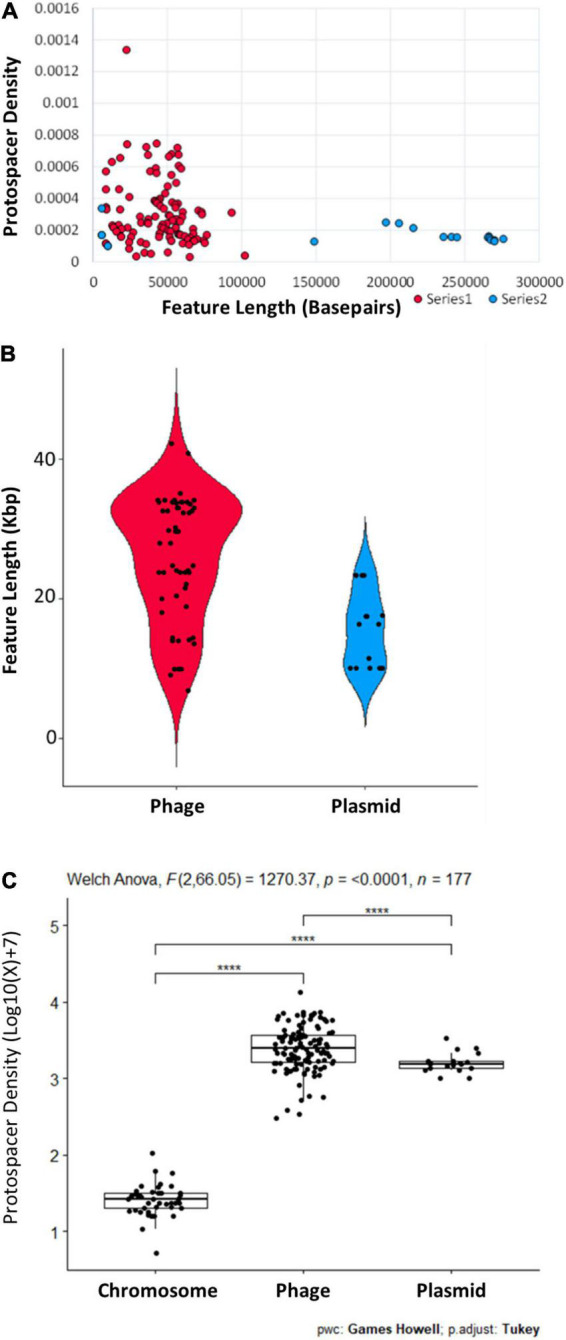
Phage and plasmids vary in length and protospacer density. **(A)** Protospacer density in phage (*n* = 115) and plasmids (*n* = 19) from 43 closed G1 *C. botulinum* and *C. sporogenes* genomes plotted relative to size in base pairs. **(B)** Range in length of phage (*n* = 55) and plasmids (*n* = 14) with no protospacer matches. **(C)** Phage and plasmids are significantly enriched in protospacers relative to the chromosome. Additional data and parameters are provided in Supplementary File 8. **** indicate statistical significance.

Initially, in terms of protospacer density, the *bont*(+) conjugative plasmids appeared to be among the least targeted plasmids present in G1 *C. botulinum* and *C. sporogenes* (Figure 4B). Investigation of the ∼40–50 kbp plasmids with ∼3–5 × greater protospacer density than the *bont*(+) plasmids suggested through the presence of numerous structural phage genes that these sequences represent circularized phage genomes, not plasmids. To more fully understand the degree to which *bont*(+) plasmids are targeted by CRISPR-Cas, we determined the protospacer density for each plasmid, phage, and the chromosome outside of prophage regions. Within the 43 strains investigated, 170 phage regions and 33 plasmids were identified (Figure 6A). No protospacers were identified in 55 phage and 14 plasmids (Figure 6B). All *bont*(+) plasmids possessed between 19 and 43 protospacer loci. A Welch one-way ANOVA of log-transformed protospacer density in chromosomal (*n* = 43), plasmid (*n* = 19), and phage (*n* = 115) revealed a statistically significant difference in protospacer density between groups: *F* = 1,270 (2,66.05), *p* ≤ 0.0001. A Ganes–Howell *post hoc* analysis revealed statistically significant differences (*p* ≤ 0.0001) in mean protospacer density between all groups (Figure 6C and Supplementary File 8). These findings indicate that plasmids, including all *bont*(+) plasmids, and phage are enriched in protospacers relative to the chromosome, indicating that uptake of the *bont* gene cluster may be impacted by CRISPR-Cas system targeting of the plasmid.

### G1 *Clostridium botulinum* and *Clostridium sporogenes* Possess a Large Shared Mobilome

**FIGURE 7:**
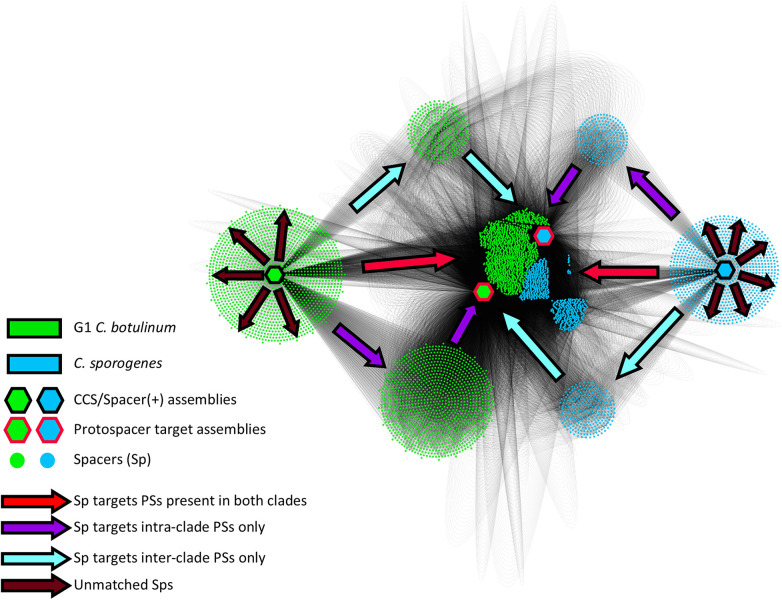
Most targeted protospacers are present in both G1 *C. botulinum* and *C. sporogenes*. Figure shows a directed matrix made in Cytoscape (Shannon, 2003) connecting CRISPR-Cas(+) assemblies (black outlined hexagonal node) to protospacers(+) (red outlined hexagonal node) through a shared intermediate node representing a unique spacer (circular node). For all spacer–protospacer interactions, each strain was collapsed into one of four clade-specific groups (G1 *C. botulinum*/*C. sporogenes* + spacer/protospacer). Both species are represented as hexagonal nodes connected to each spacer ID by edges that are 1:1 between spacer and species-spacer, and 1:X matched protospacers between spacers and protospacer-species. The arrow legend indicates spacer targets. Red arrows indicate directionality of spacers that target protospacers present in both species and represent 78% of all spacer–protospacer pairs (Table 4).

To better understand the potential relevance of these data at the population scale, we investigated the overlap of protospacers in G1 *C. botulinum* and *C. sporogenes*. Species exclusive spacer–protospacer (Sp-PS) matches (e.g., *C. sporogenes* spacers that exclusively match *C. sporogenes* protospacers) accounted for only 15.2% of total matches (Figure 7 and Table 4). Hits from one species exclusively against protospacers present in the other represented 6.8% of total hits (Figure 7 and Table 4). The vast majority, 77.6%, of matched spacers were predicted to target protospacers present in both species. These results, in conjunction with the finding that protospacer targets are predominantly prophage and plasmid associated, indicate that G1 *C. botulinum* and *C. sporogenes* possess a large shared mobilome. Analysis of viral protospacers was limited to prophage present within closed G1 *C. botulinum* and *C. sporogenes* genomes, limiting insight into the broader host range targeted bacteriophage. However, matched RefSeq plasmids from other soil-dwelling Gram-positive genera included *Paenibacillus* and *Enterococcus*, which are known to possess genes homologous to *bont* gene cluster genes (Zhang et al., 2018; Nowakowska et al., 2019). With further development, these data might enable enhanced risk assessment through the quantification of the normal range of horizontal gene transfer between G1 *C. botulinum* and other species.

**TABLE 4:** Directed force matrix.

Protospacer species	Spacer species	Spacer species	Grand totals
	*G1 C. botulinum*	*C. sporogenes*	
*G1 C. botulinum*	7,202 (G1–G1 Only)	1,840 (Cspo-G1 Only)	
*C. sporogenes*	1,975 (G1-Cspo Only)	1,309 (Cspo-Cspo Only)	
G1 [Share of G1 + *C. spo*]	17,265	9,499	
C. spo [Share of G1 + *C. spo*]	10,118	6,203	
Unmatched spacers	1,502	954	
Total shared	27,383	15,702	43,085
Total in-clade only	7,202	1,309	8,511
Total cross-clade only	1,975	1,840	3,815

## Discussion

In this study, we investigated whether CRISPR-Cas systems present in the closely related species G1 *C. botulinum* and *C. sporogenes* represent a potential barrier to the acquisition of the botulinum neurotoxin virulence factor via horizontal gene transfer. Through application of a spacer mapping and reference masking approach to predict spacers and identify cognate protospacer targets within a diverse sample of 145 G1 *C. botulinum* and 96 *C. sporogenes* strains (Figure 1), we uncovered evidence of a targeting of bacteriophage and plasmids. None of the annotated protospacers within the ∼56,000 spacer–protospacer matches occurred within the constituent *bont* gene cluster genes: *bont*, *ntnh*, *botR*, *ha17/33/70*, *p47*, and *orfX1/2/3* present in toxigenic strains in the study dataset (*n* = 154/241). Investigation of all protospacer hits within closed genomes (*n* = 43) indicated no protospacers present between *bont* gene cluster genes and none targeting nearby group II introns and ISs. As of today, no IS elements near the *bont* gene clusters have been shown to be functional. Emerging research will further elucidate their potential role in the transfer of *bont* gene clusters and their (non)targeting by restriction systems such as CRISPR-Cas. Intriguingly, the IS200/IS605 IS elements, the only IS family with matched spacers by CRISPR-Cas systems in our study, have themselves been recently demonstrated to be programmable RNA-guided nucleases related to the type V CRISPR-Cas system effector (Cas12) (Altae-Tran et al., 2021). Functional characterization of these ISs alongside the endogenous CRISPR-Cas systems is required to determine whether this targeting represents competition or collaboration in these two species. The absence of targeting of other IS elements supports the hypothesis that IS elements play a beneficial role to the bacterial host in acquiring and retaining potentially beneficial gene(s) such as the *bont* gene cluster. However, targeting of IS element activity by an alternate mechanism such as other restrictive processes, such as RM systems, cannot be excluded based on our data.

Approximately 80% of the protospacer targets of CRISPR-Cas systems in G1 *C. botulinum* and *C. sporogenes* were present across both species, indicating these species possess a large shared mobilome (Figure 7). Through protospacer inference, the CRISPR-Cas system targeted mobilome predominantly consists of bacteriophage and to a lesser extent plasmids (Figures 5, 6). These findings run contrary to a previous report of more frequent matches between spacers and plasmid associated protospacers than phage associated protospacers in *C. botulinum* (Biswas et al., 2013; Negahdaripour et al., 2017; Brooks et al., 2019). These differences are explained by differing methodology, as GenBank-Phage used in that study to search for protospacers only identified sequenced bacteriophage present in GenBank-Phage while the mapping approach utilized in this study enabled identification of all annotated bacteriophage within the chromosome (Rohwer, 2003; Hatfull and Hendrix, 2011; Biswas et al., 2013; Arndt et al., 2016). Our observed association of 61% of protospacers with bacteriophage approaches the 70–90% rate reported in most bacterial species (Shmakov et al., 2017). This likely still represents an undercount of phage associated protospacers, as examination of protospacer annotations (e.g., phage tail family protein) at other chromosomal sites (15%) suggests the presence of phage remnants or non-annotated phage (Supplementary File 7).

We observed a clear bifurcation in protospacer density within G1 *C. botulinum* and *C. sporogenes* plasmids relative to those observed in other species (Figure 4B). Relative to a previous study that generated spacer–protospacer matches utilizing blastN within the CRISPR-Target program and RefSeq plasmids (Biswas et al., 2013; Negahdaripour et al., 2017; Brooks et al., 2019), we observed lower diversity in the protospacer containing plasmids sequenced in other genera. While the reference mapping settings utilized in our study allowed identification of protospacers with up to two mismatches and local alignment allowed some flexibility at the ends of the spacer-protospacer alignment, this approach is conservative compared with an 80% identity blastN threshold (Negahdaripour et al., 2017) and may have excluded more distant matches in other genera. Interestingly, nearly all matched plasmids were Gram-positive bacteria, and hits to plasmids in genera including *Enterococcus* and *Paenibacillus* are consistent with rare, but documented horizontal gene flows of *bont* gene cluster constituents (Zhang et al., 2018; Nowakowska et al., 2019). CRISPR-Cas system spacers will generally target MGEs that are most often encountered by the host strains (Shmakov et al., 2017), indicating that the plasmids present in the G1 *C. botulinum* and *C. sporogenes* mobilomes have been present long enough for CRISPR-CAS system mediated immunity to develop in certain strains. This also indicates plasmid targeting occurs, and CRISPR-Cas systems may constitute a barrier to uptake in the minority of analyzed strains possessing a functional CRISPR-Cas system and the appropriate spacer(s) (Figure 4C). For example, *C. sporogenes* PA 3679 (genetic G1 *C. botulinum*) possess spacers against several *bont*(+) plasmids, which could limit conjugation of *bont*(+) plasmids into this strain, and by extension acquisition of toxicity. However, this would be achieved through targeting of the vehicle, not the toxin. The cross-strain variation in CRISPR-Cas systems also suggests they are unlikely to play a major restrictive role regulating toxin transfer at the species or bi-species level.

Type I-B, III-B, and III-D CRISPR-Cas systems have been identified in (G1–G3) *C. botulinum* by prior studies utilizing closed genomes, with type III-B CRISPR-Cas systems reported as the most prevalent (Hatoum-Aslan and Marraffini, 2014; Negahdaripour et al., 2017; Puigbò et al., 2017). Our study utilized both closed and contig-level genomes extended these findings to neighbor species *C. sporogenes* and additionally identified the presence of III-A CRISPR-Cas systems in both species. Through identification of conserved genomic markers and utilization of verified and predicted plasmids, we were able to determine exclusive chromosomal localization of the type III systems and identified key differences in the *cas* gene composition of chromosomal and plasmid type I-B CRISPR-Cas systems (Figures 2B,C). These additional findings are the result of study design, scope, and time elapsed since the previous studies were conducted (Hatoum-Aslan and Marraffini, 2014; Negahdaripour et al., 2017; Puigbò et al., 2017). For example, utilization of the RFPlasmid program (Van Bloois et al., 2020) enabled the identification of several non-canonical variants of the type I-B CRISPR-CAS system. In contrast to the observed presence/absence and pseudogenization of individual type III genes across strains, potentially indicating a loss of function, chromosomal type I-B systems were rarely pseudogenized. The exclusive and mostly conserved presence of a nuclease(−) type I-B* on *bont*(+) conjugative plasmids may indicate degraded or functionally atypical CRISPR-Cas system. Recently, a CRISPR-Cas mediated toxin–antitoxin system was linked to the retention of effector gene function in type I-B systems in archaea and some bacterial species (Li et al., 2021). A similar mechanism could explain the persistence of the plasmid-borne type I-B* systems consisting of only *cas6* and the *cas5/7/8* effectors. In addition, the type I-B* CRISPR-Cas system also appears to have the *cas* gene set necessary to perform a CRISPRi type function, which have been proposed to potentially play a regulatory role (Vial and Hommais, 2020; Wimmer and Beisel, 2020). There is also some similarity between the type I-B* CRISPR-Cas system and a distinct group of type I-F CRISPR-Cas systems in *Vibrio* spp. transposons that lack a *cas3* nuclease gene and possess *cas6*, *cas7*, and a *cas5*/8 fusion genes and are utilized to achieve CRISPR-mediated site-specific transposition within the genome (Peters et al., 2017; Klompe et al., 2019; Mcdonald et al., 2019). The integrated *bont*(+) plasmids (Dover et al., 2014; Smith et al., 2021a) observed at integration sites 1 and 4 are intriguing within that context and suggest that much remains to be learned about these still cryptic plasmids (Figure 3B). Direct functional investigation and characterization of this unique, *bont*(+) plasmid exclusive, type I-B system will provide additional insight into its function and potential relationship to the *bont* gene cluster.

CRISPR-Cas systems are dynamic recombination sites, which makes accurate identification of CRISPR Cas systems and *cas* types challenging. *C. botulinum* B1 Okra was reported to possess a chromosomal type III-B and plasmid-borne I-B CRISPR-Cas systems (Negahdaripour et al., 2017), while another study from the same year identified that same strain as possessing a III-D CRISPR-Cas system and highlighting it as an example of a recombination event whereby the III-B CRISPR-Cas system was supplanted by a III-D CRISPR-Cas system with *cas6* remaining unaffected (Kristensen et al., 2017). Our data also showed phylogenetic co-clustering of the type III-B and III-D associated Cas6 proteins, which is consistent with potential recombination between type III-B and III-D CRISPR-Cas systems. However, our study also discovered the presence of type III-A CRISPR-Cas systems in several strains, with the associated Cas6 not phylogenetically grouping with type III-B and III-D associated Cas6 proteins (Supplementary Figure 1). Detailed examination of gene gain/loss falls beyond the scope of this study; however, the data collected potentially lends itself to such analysis in future studies. For example, we observed evidence that the type I-B system within site 1 occurs in a minority of both *C. botulinum* and *C. sporogenes* strains (Supplementary Figure 1 and Supplementary File 5). A blast search shows that *C. tepidum*, the nearest neighbor species to G1 *C. botulinum* and *C. sporogenes* (Dobritsa et al., 2017), also possesses a homologous type I-B CRISPR-Cas system at the same location (NZ_JADPGM010000006.1). This could indicate either that the common ancestor of all three species possessed a I-B CRISPR-Cas system at site 1 (vertical heritage) or that the I-B integration at site 1 has occurred independently multiple times (horizontal acquisition). The presence of pseudogenized *cas6* genes and broad presence of orphan CRISPR features throughout both species would support vertical heritage while the relative scarcity of the type I-B CRISPR-Cas system at site 1 would support horizontal acquisition. Understanding the dynamics of additional acquisition of genes at sites beyond the BoNT gene clusters will enable deeper investigation of how these hypervariable genomic regions are governed.

The results of our study have revealed broad similarities between G1 *C. botulinum* and *C. sporogenes* in both the types of CRISPR-Cas systems present and the mobile targets that they defend against. A recent pan-genomic analysis by Brunt and colleagues found that unique genes to G1 *C. botulinum* and *C. sporogenes* map regularly throughout the length of the genome with no identifiable hotspots (Brunt et al., 2020a). This is consistent with findings in this study that (1) the prophage and plasmids that make up the bulk of the CRISPR-Cas system targeted mobilome are predominantly shared between G1 *C. botulinum* and *C. sporogenes*, and (2) the chromosomal locations of hypervariable sites/hotspots seem to be shared across the two species. In future comparative genomic studies of recombination and integration at hypervariable sites, it may prove beneficial to consider both species in the context of additional species outgroups such as *C. tepidum*. In particular, additional closed genomes from a broader and more diverse range of strains will provide further insight into the regulation of these sites and the selective pressures that enable these sites to acquire, host, and eliminate sophisticated genomic defense modules, the most potent known biological toxin, or nothing at all.

Our systematic investigation of CRISPR-Cas systems in G1 *C. botulinum* and *C. sporogenes* revealed a predominantly shared mobilome between these neighboring species and widespread (∼83%) presence of CRISPR-Cas system features across strains of both. However, the capacity to utilize the adaptive immune component of CRISPR-Cas systems was present in only 16% of strains with chromosomally localized type I-B CRISPR-Cas systems, and the plasmid exclusive presence of partial type I-B systems presents the possibility that plasmids, including the family that carries the *bont* gene cluster, are utilizing CRISPR-Cas with some degree of autonomy from the host. Inclusion of contig level genomes did present analytical challenges. For example, we did not systematically predict PAM sequences associated with the type I-B systems as it was often challenging to associate CRISPR arrays and *cas* gene clusters in contig-level assemblies. PAM determination would be best achieved on closed subsets and ideally in the context of functional characterization of the CRISPR-Cas systems. However, inclusion of contig level genomes ultimately led to a larger, more diverse spacer set than would have been obtainable through closed genomes alone.

Taken together, our data show that despite being the most prominent horizontally trafficked gene cluster in *Clostridium*, the *bont* gene cluster was not directly targeted by the endogenous CRISPR-Cas systems of G1 *C. botulinum* and *C. sporogenes*. However, these systems do appear to target the conjugative plasmids that traffic the *bont* gene clusters in certain G1 *C. botulinum* and *C. sporogenes* strains. Future functional investigation of the diverse endogenous CRISPR-Cas systems in both species will provide further insight into the regulation of these shared dynamic genomic regions host to both complementary genomic defense systems and the most potent known bacterial toxin.

## Data Availability Statement

The original contributions presented in the study are included in the article/Supplementary Material, further inquiries can be directed to the corresponding author/s.

## Author Contributions

TW, AD, J-DS, and SP conceived and designed the research. TW acquired and interpreted the data. TW, BT, and MB analyzed the data. TW wrote the manuscript. TW, BT, MB, AD, SS, J-DS, and SP contributed to article revision, read and approved the submitted version, and provided approval for the publication of the content. All the authors contributed to the article and approved the submitted version.

## Conflict of Interest

The authors declare that the research was conducted in the absence of any commercial or financial relationships that could be construed as a potential conflict of interest.

## Publisher’s Note

All claims expressed in this article are solely those of the authors and do not necessarily represent those of their affiliated organizations, or those of the publisher, the editors and the reviewers. Any product that may be evaluated in this article, or claim that may be made by its manufacturer, is not guaranteed or endorsed by the publisher.

## Footnotes

1 Seemann T, mlst Github https://github.com/tseemann/mlst

## Supplementary Material

Supplementary Figure 1 Cas6 protein alignment and phylogeny. Multiple alignment (Clustal Omega Default) (Sievers et al., 2011) and phylogenetic analysis (raxml -PROTGAMMAAUTO) (Stamatakis, 2014) of all Cas6 proteins identified within the study dataset indicates divergence between the type I-B systems present at genomic site 1, site 4, and plasmid-borne variants (Supplementary File 5). Type III-B and III-D Cas6 form a single, high identity branch, while type III-A Cas6 proteins load on separate branches. All Cas6 loci are listed in Supplementary File 6.

Supplementary Figure 2 Plasmid-borne *bont* gene clusters occur on a family of related conjugative plasmids. Plasmid pNPD7 and the putative integrated plasmid from *C. sporogenes* CDC 67071 share the conserved conjugation region present in most G1 *C. botulinum bont*(+) conjugative plasmids. Local alignment (blastN) of select *bont*(+) plasmids against pCBG; with a type I-B* CRISPR-Cas system present at S5, a *bont/A* gene cluster at S6, and *bont/B* gene cluster at S7. A region of chromosome from *C. botulinum* IBCA10-7060 from 2339765–2389948 and a *bont*(−) conjugative plasmid pRSJ2_2 (NZ_CP013709.1) were additionally included. BRIG was run with pCBG set as the reference with the following settings: blastN, 70% upper identity threshold, 50% lower identity threshold, and annotated with site information (Alikhan et al., 2011). The *bont* genes and the type I-B* gene clusters from pCBG are highlighted (red) within plasmid sites 5, 6, and 7 (gray) as defined in Figure 3. **(A)** The type I-B* CRISPR-Cas systems are present on all family plasmids except a subset of *bont/A, B* producing plasmids such as pCLJ. **(B)** The plasmid fragment adjacent to chromosomal *bont/FA(H)* at site 4 in strain (Figure 3) is part of an insert unique to *bont/A, B* plasmids. **(C)** The *bont/B1* plasmid is ∼100 kbp shorter than other family members and is missing the region including the putative conjugal type IV secretion system. **(D)** The *bont*(−) ∼200-kbp plasmids carrying the type I-B^**^ CRISPR-Cas system are unrelated to *bont*(+) 250-kbp plasmids.

Supplementary Figure 3 Full-length and/or fragmented group II introns near select subtypes of *bont/A*, *B*, and *F* gene clusters. **(A)** Local alignment (blastN default) of the plasmid-borne *bont*/A3 gene cluster in *C. botulinum* A3 Loch Maree against the chromosomal *bont*/A3 gene cluster in *C. botulinum* 1169. The CDS for the putative group II intron encoded protein (IEP) is annotated as *ltrA*. **(B)** Predicted secondary structure of the group II intron surrounding CDS ltrA in strain A3 Loch Maree with conserved domains (I–VI) labeled. **(C)** Location of a *ltrA* fragment present at the *arsC* disruption site in a subset of chromosomally integrated *bont*/A and F gene clusters. **(D)** Multiple alignment of full-length *ltrA* genes and *ltrA* fragments from representative strains (Clustal omega, default settings). **(E)** Phylogenetic distribution of *ltrA* fragments (filled blue circle) and full-length *ltrA* genes (filled red square) across the study strains. *Primary hit: *E* value 0.0, 86% coverage, 99.08% identity, hits ≤ *E* value 1e-50 displayed.
