# Supplementary figures and images for: Endogenous CRISPR-Cas Systems in Group I Clostridium botulinum and Clostridium sporogenes Do Not Directly Target the Botulinum Neurotoxin Gene Cluster
Source: Front Microbiol. 2022 Feb 9;12:787726. doi: 10.3389/fmicb.2021.787726 (PMC8865420; doi:10.3389/fmicb.2021.787726)

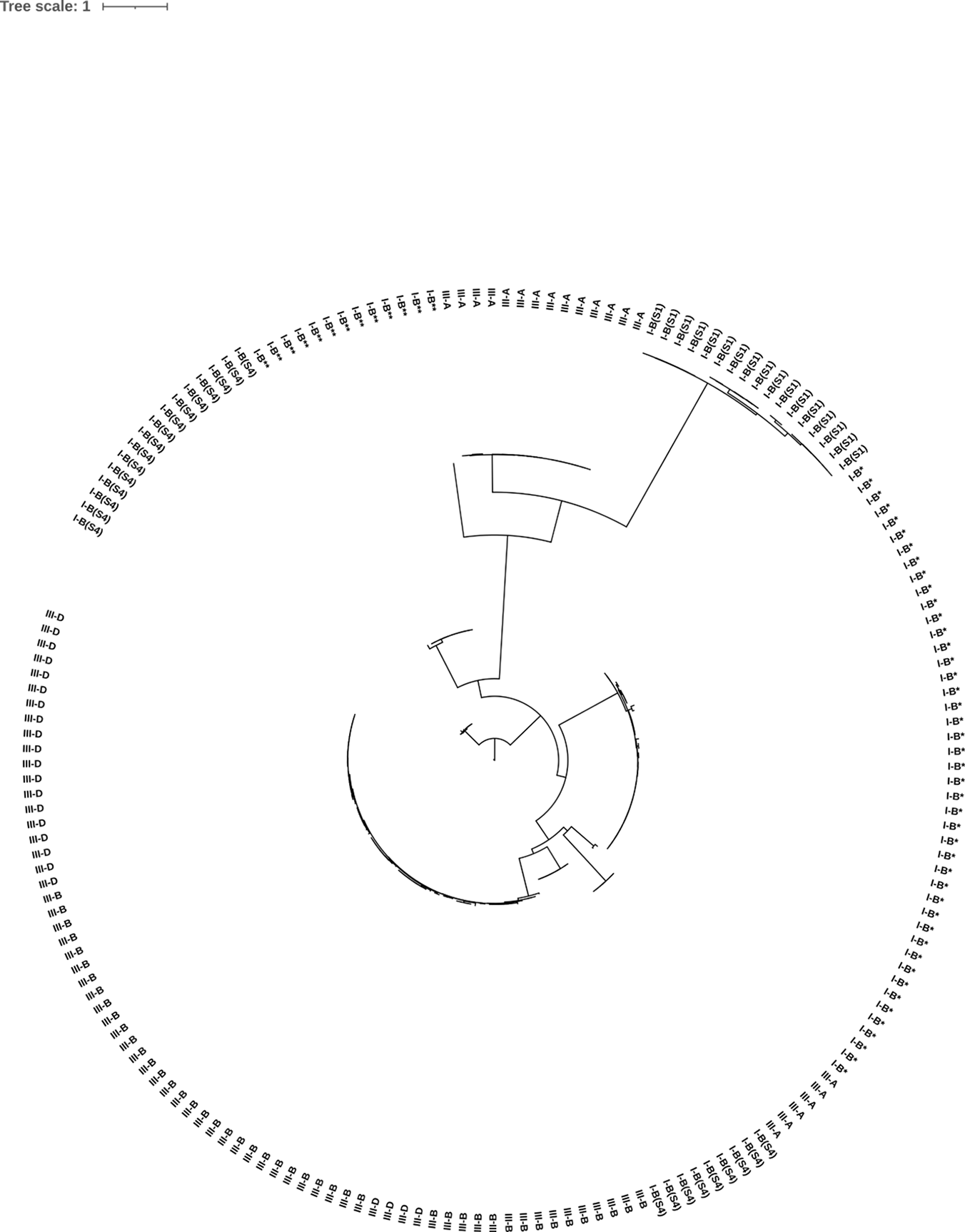

Supplement: Supplementary Figure 1 — Cas6 protein alignment and phylogeny. Multiple alignment (Clustal Omega Default) (Sievers et al., 2011) and phylogenetic analysis (raxml -PROTGAMMAAUTO) (Stamatakis, 2014) of all Cas6 proteins identified within the study dataset indicates divergence between the type I-B systems present at genomic site 1, site 4, and plasmid-borne variants (Supplementary File 5). Type III-B and III-D Cas6 form a single, high identity branch, while type III-A Cas6 proteins load on separate branches. All Cas6 loci are listed in Supplementary File 6. [file Image_1.TIF]

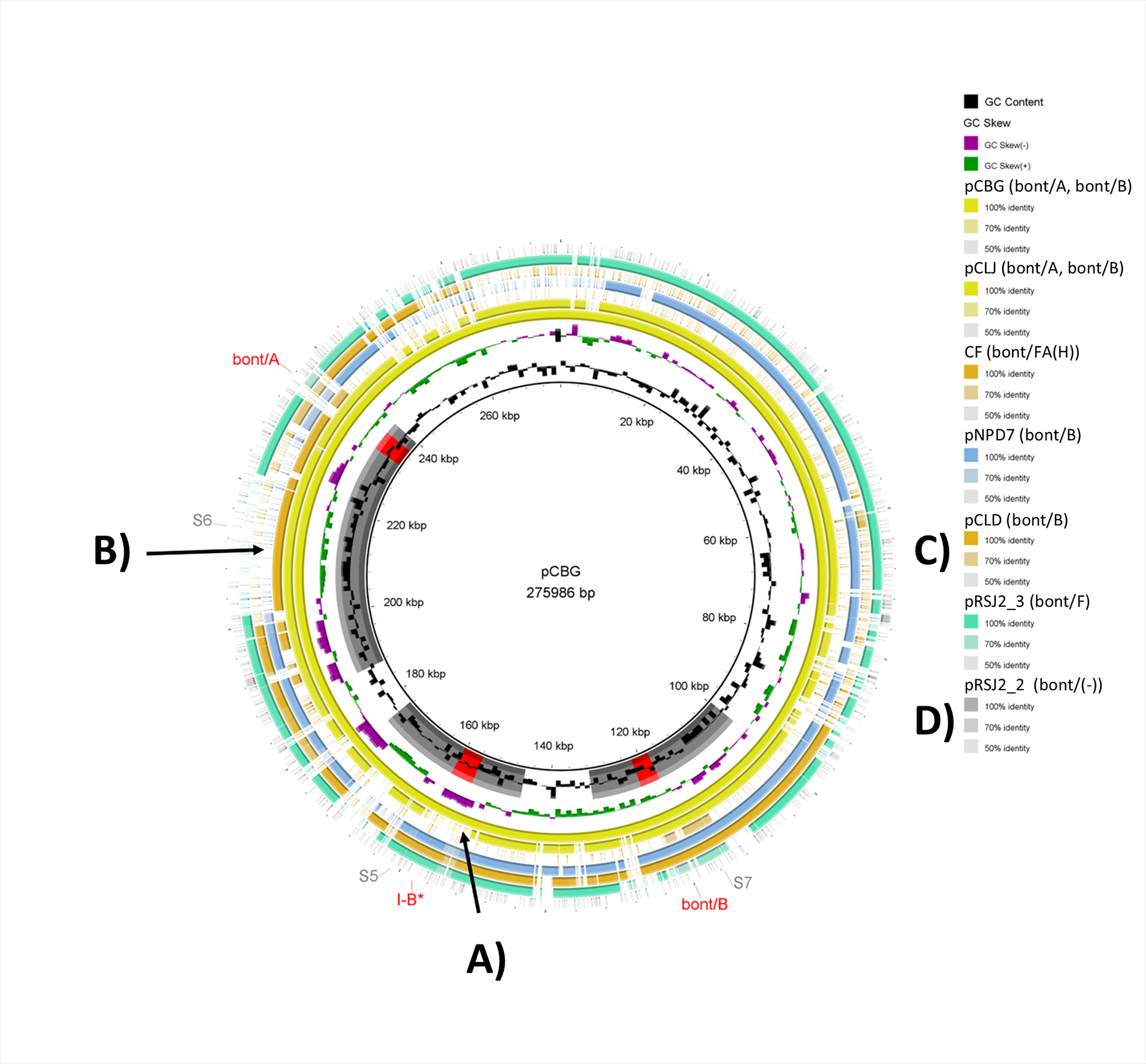

Supplement: Supplementary Figure 2 — Plasmid-borne bont gene clusters occur on a family of related conjugative plasmids. Plasmid pNPD7 and the putative integrated plasmid from C. sporogenes CDC 67071 share the conserved conjugation region present in most G1 C. botulinum bont(+) conjugative plasmids. Local alignment (blastN) of select bont(+) plasmids against pCBG; with a type I-B* CRISPR-Cas system present at S5, a bont/A gene cluster at S6, and bont/B gene cluster at S7. A region of chromosome from C. botulinum IBCA10-7060 from 2339765–2389948 and a bont(−) conjugative plasmid pRSJ2_2 (NZ_CP013709.1) were additionally included. BRIG was run with pCBG set as the reference with the following settings: blastN, 70% upper identity threshold, 50% lower identity threshold, and annotated with site information (Alikhan et al., 2011). The bont genes and the type I-B* gene clusters from pCBG are highlighted (red) within plasmid sites 5, 6, and 7 (gray) as defined in Figure 3. (A) The type I-B* CRISPR-Cas systems are present on all family plasmids except a subset of bont/A, B producing plasmids such as pCLJ. (B) The plasmid fragment adjacent to chromosomal bont/FA(H) at site 4 in strain (Figure 3) is part of an insert unique to bont/A, B plasmids. (C) The bont/B1 plasmid is ∼100 kbp shorter than other family members and is missing the region including the putative conjugal type IV secretion system. (D) The bont(−) ∼200-kbp plasmids carrying the type I-B** CRISPR-Cas system are unrelated to bont(+) 250-kbp plasmids. [file Image_2.TIF]

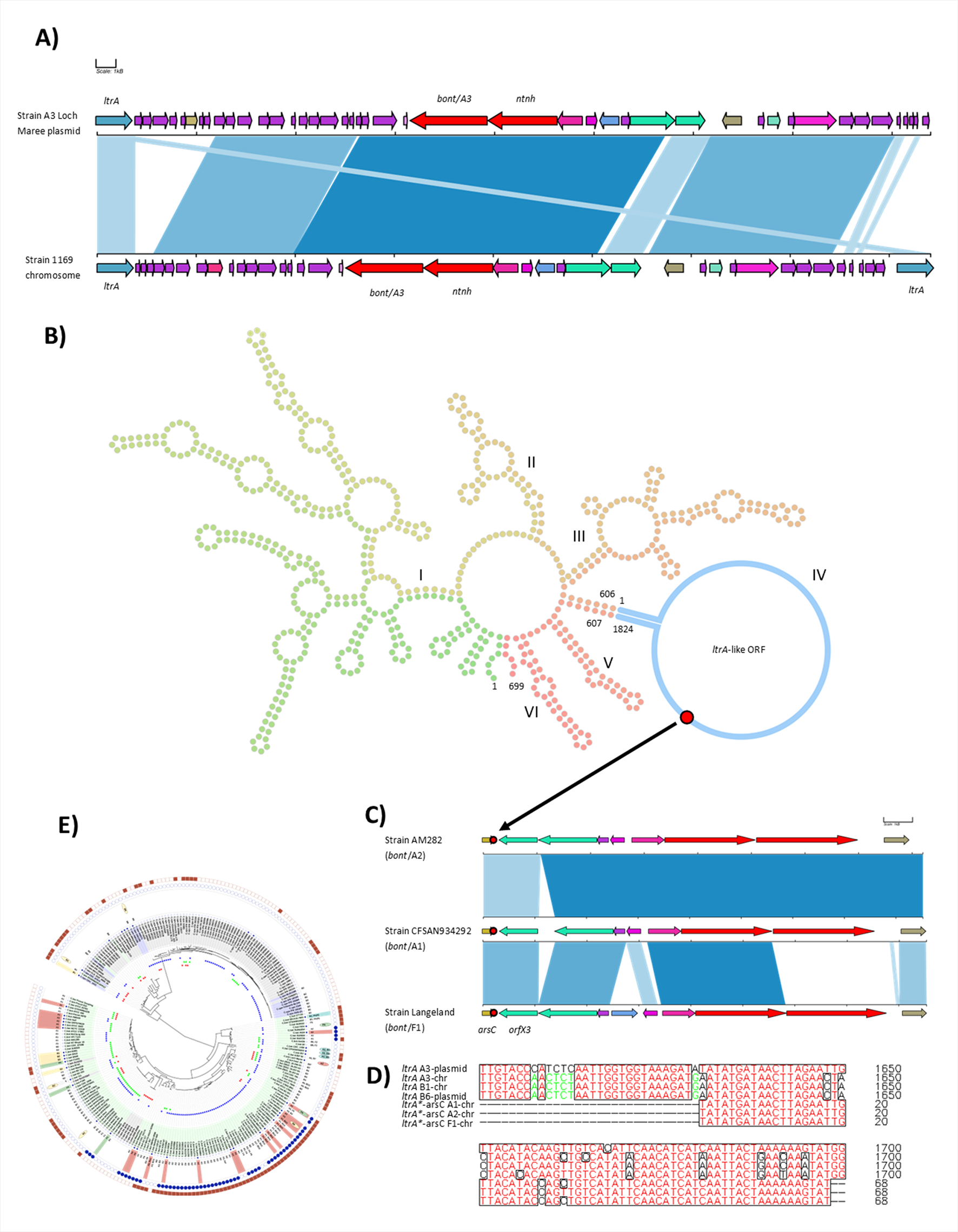

Supplement: Supplementary Figure 3 — Full-length and/or fragmented group II introns near select subtypes of bont/A, B, and F gene clusters. (A) Local alignment (blastN default) of the plasmid-borne bont/A3 gene cluster in C. botulinum A3 Loch Maree against the chromosomal bont/A3 gene cluster in C. botulinum 1169. The CDS for the putative group II intron encoded protein (IEP) is annotated as ltrA. (B) Predicted secondary structure of the group II intron surrounding CDS ltrA in strain A3 Loch Maree with conserved domains (I–VI) labeled. (C) Location of a ltrA fragment present at the arsC disruption site in a subset of chromosomally integrated bont/A and F gene clusters. (D) Multiple alignment of full-length ltrA genes and ltrA fragments from representative strains (Clustal omega, default settings). (E) Phylogenetic distribution of ltrA fragments (filled blue circle) and full-length ltrA genes (filled red square) across the study strains. *Primary hit: E value 0.0, 86% coverage, 99.08% identity, hits ≤ E value 1e-50 displayed. [file Image_3.TIF]
