# Supplementary material for: Endogenous CRISPR-Cas Systems in Group I Clostridium botulinum and Clostridium sporogenes Do Not Directly Target the Botulinum Neurotoxin Gene Cluster
Source: Front Microbiol. 2022 Feb 9;12:787726. doi: 10.3389/fmicb.2021.787726 (PMC8865420; doi:10.3389/fmicb.2021.787726)
Supplement: Supplementary file 4 [file Data_Sheet_1.docx]

Supplementary Tables

| Pangenomic summary statistics | | | |
| --- | --- | --- | --- |
| Composition | Core Genes | Accessory Genes | Total |
| 250 strains (Passing MLST) | 1,840 | 12,523 | 14,363 |
| 241 strains (Study Dataset) | 2,003 | 11,840 | 13,843 |
| G1 *C. botulinum* clade  -139 C. botulinum (2 non-toxic)  -1 *C. combesii*  -5 *C. sporogenes* (PA3679) | 2,101 | 8,611 | 10,712 |
| C. sporogenes clade  -89 *C. sporogenes*  -7 *C. botulinum* (4 non-toxic) | 2,030 | 7,937 | 9,967 |

Supplementary Table 1: Summary statistics from pan-genomic analysis of *C. sporogenes* and *C. botulinum*.

**
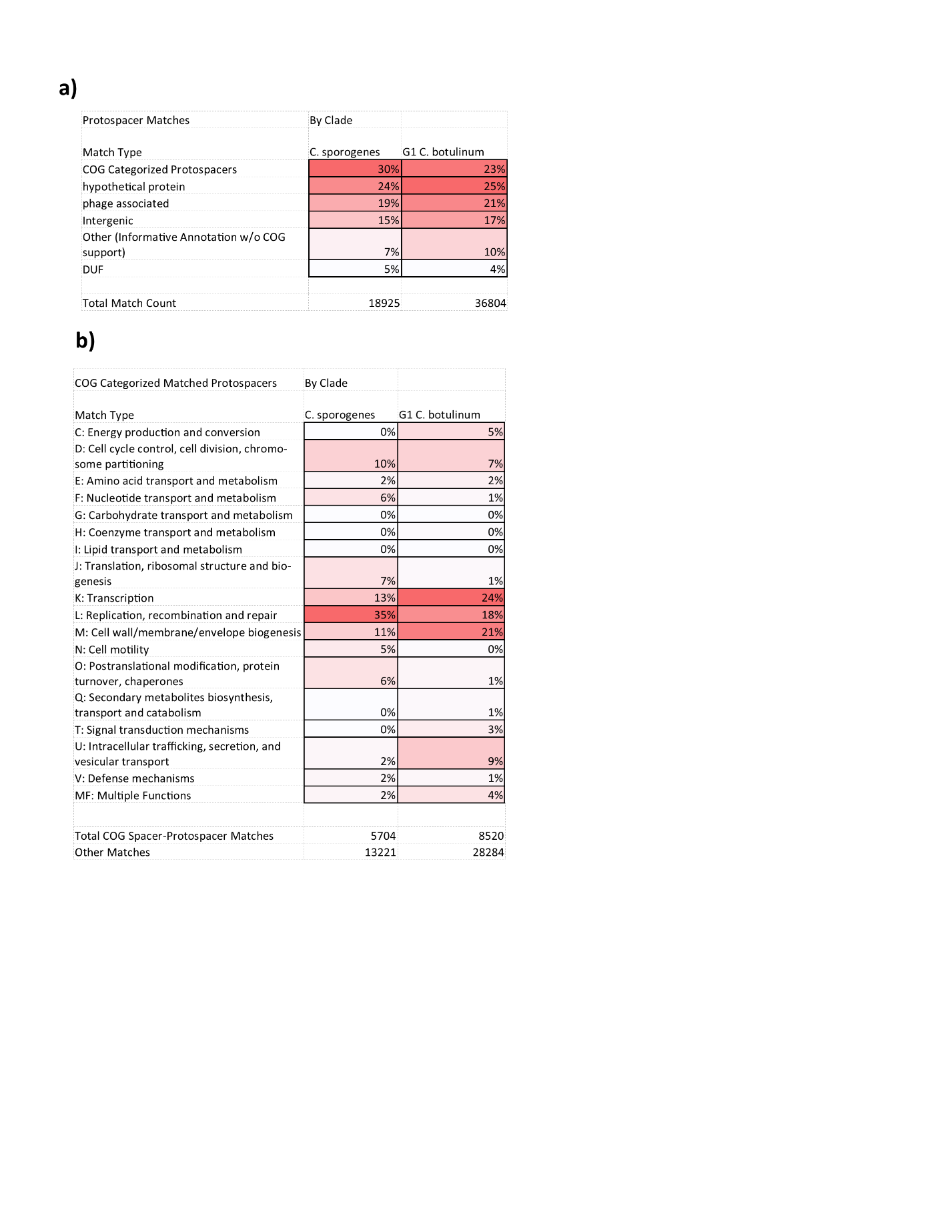
**

Supplementary Table 2: 2a) Characterization of matched protospacers by spacer species or associated *cas* gene cluster. 2b) Characterization of matched protospacers by COG function.
